# Studying the Potential Effects of Artificial Intelligence on Physician Autonomy: Scoping Review

**DOI:** 10.2196/59295

**Published:** 2025-03-13

**Authors:** John Grosser, Juliane Düvel, Lena Hasemann, Emilia Schneider, Wolfgang Greiner

**Affiliations:** 1 Department of Health Economics and Health Care Management School of Public Health Bielefeld University Bielefeld Germany; 2 Centre for Electronic Public Health Research (CePHR) School of Public Health Bielefeld University Bielefeld Germany

**Keywords:** autonomy, professional autonomy, physician autonomy, ethics, artificial intelligence, clinical decision support systems, CDSS, ethics of artificial intelligence, AI ethics, AI, scoping review, physician, acceptance, adoption

## Abstract

**Background:**

Physician autonomy has been found to play a role in physician acceptance and adoption of artificial intelligence (AI) in medicine. However, there is still no consensus in the literature on how to define and assess physician autonomy. Furthermore, there is a lack of research focusing specifically on the potential effects of AI on physician autonomy.

**Objective:**

This scoping review addresses the following research questions: (1) How do qualitative studies conceptualize and assess physician autonomy? (2) Which aspects of physician autonomy are addressed by these studies? (3) What are the potential benefits and harms of AI for physician autonomy identified by these studies?

**Methods:**

We performed a scoping review of qualitative studies on AI and physician autonomy published before November 6, 2023, by searching MEDLINE and Web of Science. To answer research question 1, we determined whether the included studies explicitly include physician autonomy as a research focus and whether their interview, survey, and focus group questions explicitly name or implicitly include aspects of physician autonomy. To answer research question 2, we extracted the qualitative results of the studies, categorizing them into the 7 components of physician autonomy introduced by Schulz and Harrison. We then inductively formed subcomponents based on the results of the included studies in each component. To answer research question 3, we summarized the potentially harmful and beneficial effects of AI on physician autonomy in each of the inductively formed subcomponents.

**Results:**

The search yielded 369 studies after duplicates were removed. Of these, 27 studies remained after titles and abstracts were screened. After full texts were screened, we included a total of 7 qualitative studies. Most studies did not explicitly name physician autonomy as a research focus or explicitly address physician autonomy in their interview, survey, and focus group questions. No studies addressed a complete set of components of physician autonomy; while 3 components were addressed by all included studies, 2 components were addressed by none. We identified a total of 11 subcomponents for the 5 components of physician autonomy that were addressed by at least 1 study. For most of these subcomponents, studies reported both potential harms and potential benefits of AI for physician autonomy.

**Conclusions:**

Little research to date has explicitly addressed the potential effects of AI on physician autonomy and existing results on these potential effects are mixed. Further qualitative and quantitative research is needed that focuses explicitly on physician autonomy and addresses all relevant components of physician autonomy.

## Introduction

The use of artificial intelligence (AI) systems in medicine has increased significantly in recent years. AI in medicine can take a number of forms and fulfill a number of tasks, ranging from risk prediction or diagnosis and screening to AI-powered clinical decision support systems (CDSS) [1]. AI systems have also been introduced across a range of medical specialties, including oncology, pulmonology, and radiology [2].

Physician autonomy has been found to play a role in physician acceptance and adoption of medical technologies [3], and in particular, AI [1]. Although physician autonomy has become an increasingly important concept in recent decades [4-7], there is still no consensus definition in the literature. However, physician autonomy is generally seen as including both clinical freedoms, as well as social and economic freedoms [6,7]. The former concerns physician autonomy in clinical practice, including their control over the diagnosis and treatment of patients and over evaluations of their care. The latter concerns the autonomy of physicians as professionals, including their choice of specialty and control over the nature and volume of their tasks [5]. A number of recent reviews have found that the feared loss of physician autonomy represents a barrier to the acceptance of AI [1,8-10]. However, although these reviews (partially) address physician autonomy as a barrier to acceptance, there is little research so far focusing primarily on the effects of AI on physician autonomy. Furthermore, such reviews rarely systematically address both clinical, social, and economic freedoms.

Our aim is to begin to fill this gap by performing a scoping review of qualitative studies on AI and physician autonomy. In particular, this review addresses the following research questions: (1) How do these studies conceptualize and assess physician autonomy? (2) Which aspects of physician autonomy are addressed by these studies? (3) What are the potential benefits and harms of AI for physician autonomy identified by these studies? To address research question 1, we investigate whether and how the studies include physician autonomy as a research focus in their interview, survey, and focus group questions. To answer research question 2, we identify the components of physician autonomy addressed by the studies based on the 7-component model proposed by Schulz and Harrison [5]. For each of these components, we then inductively form subcomponents based on the results of the included studies. To answer research question 3, we summarize the potential benefits and harms of AI for physician autonomy reported by the included studies in each subcomponent. These questions lend themselves to a scoping review approach, rather than a systematic review since we aim to answer broader conceptual and methodological questions, rather than perform a risk of bias assessment or meta-analysis [11].

## Methods

### Search Strategy

We performed a scoping review of qualitative studies on AI and physician autonomy and drafted the paper according to the PRISMA (Preferred Reporting Items for Systematic Reviews and Meta-Analyses) checklist (Multimedia Appendix 1) [11]. We searched MEDLINE and Web of Science using a search string based on the following combination of concepts: “Physician” AND “Artificial Intelligence” AND “Autonomy” AND “Qualitative Research.” The complete search terms for both databases (including Medical Subject Headings terms and keywords) can be found in Multimedia Appendix 2. The cutoff date for the search was November 6, 2023.

### Screening

After removing duplicates, the titles and abstracts of the remaining studies were screened by 2 authors (JD and LH) according to predefined inclusion and exclusion criteria (Textbox 1). This was followed by a screening of the remaining full texts. Disagreements and concerns regarding the results were resolved in consultation with a third researcher (JG).

**Inclusion and exclusion criteria.**

**Inclusion criteria**

- Empirical, qualitative, or mixed methods study
- Focus on artificial intelligence (AI) in clinical care
- Physician autonomy addressed in the study
- The study population includes physicians
- English or German language

**Exclusion criteria**

- Nonempirical or purely quantitative study
- No focus on AI
- Focus on AI in veterinary medicine or public health
- Physician autonomy not addressed in the study
- The study population does not include physicians
- Language other than English or German

### Data Extraction and Synthesis

For each included study, we first extracted relevant study characteristics, including country, design, and study population, as well as the AI system under consideration. We also ascertained whether the included studies explicitly include physician autonomy as a research focus and reviewed supplemental material, where available, to determine whether their interview, survey, and focus group questions explicitly name physician autonomy or implicitly include aspects of physician autonomy. We then extracted the qualitative results of the studies, categorizing them into 7 components of physician autonomy introduced by Schulz and Harrison [5]. This categorization contains 3 social and economic freedoms (Textbox 2) and 4 clinical freedoms (Textbox 3).

**Social and economic components of physician autonomy [5].**

**Choice of specialty and practice location**

- Potential limitations on autonomy include market restrictions, bureaucratic restrictions, and educational restrictions

**Control over earnings**

- Potential limitations on autonomy include workload controls, fee schedules, reimbursement rates, salaried status, and control over permitted earnings

**Control over the nature and volume of medical tasks**

- Potential limitations on autonomy include hierarchical management, contractual obligations, and the need to share scarce resources

**Clinical components of physician autonomy [5].**

**Acceptance of patients**

- Potential limitations on autonomy include compelling physicians to accept or reject certain patients based on geography, medical specialty, or insurance status

**Control over diagnosis and treatment**

- Potential limitations on autonomy include individual and aggregate constraints on tests or prescription costs, preset budgets, enforcement of clinical protocols, and gatekeeping

**Control over evaluation of care**

- Potential limitations on autonomy include peer review, medical audit systems, and comparative information on care outcomes

**Control over other professionals**

- Potential limitations on autonomy include limitations on physicians’ ability to directly manage other health professionals and include precise instructions in referrals for diagnosis or therapy

To paint a more detailed picture of the effect of AI on physician autonomy, we inductively formed subcomponents from the results in each component. To avoid overgeneralizing based on individual participants and studies, we only considered subcomponents that were addressed by at least 2 included studies. Finally, we summarized the potentially harmful and beneficial effects of AI on physician autonomy in each of the inductively formed subcomponents.

## Results

### Selection of Sources of Evidence

**Figure 1:**
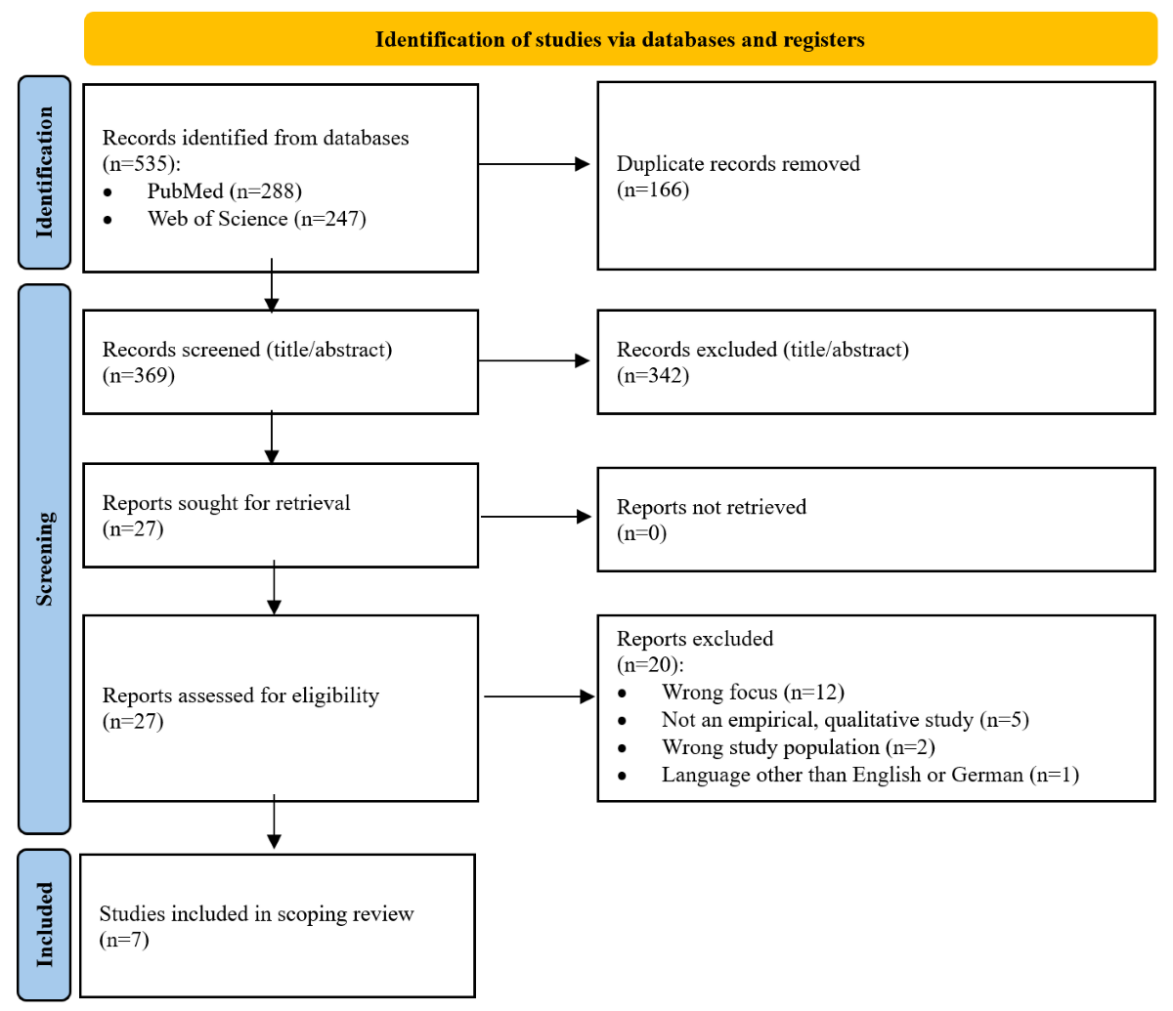
Flowchart showing the selection of sources of evidence.

The search yielded 369 studies after duplicates were removed (Figure 1). Of these, 27 studies remained after titles and abstracts were screened. After full texts were screened, we included a total of 7 qualitative studies [12-18].

### Study Characteristics

All 7 included studies had a cross-sectional design; most studies (n=5) used (qualitative) semistructured interviews, which 1 study [13] combined with a focus group. The remaining studies used co-design workshops [16] and a mixed methods survey consisting of both quantitative and qualitative items [15] (although we focus only on the qualitative results). More than half of the studies (n=4) were conducted in Europe; 2 studies were conducted in Asia and one in Australia (Table 1). Radiologists [13,17] and general practitioners (GPs) or primary care physicians [16,18] were the focus of 2 studies each, while the remaining studies recruited participants across multiple specialties. Some studies also included further groups, such as patients or family members [12,18], medical students [15], and radiographers [13], in addition to physicians. The most common form of (medical) AI investigated was CDSS (n=3). Digital disease surveillance systems and documentation assistants were investigated by 1 study each. The remaining 2 studies investigated various forms of AI in medicine. However, only 1 study [17] explicitly recruited participants who had experience with medical AI systems; the remaining studies merely provided participants with vignettes or videos of possible AI systems.

**Table 1:** Study characteristics of the included studies.

Study	Country	Study period	Participants	AI^a^ system
Amann et al (2023) [12]	Germany and Switzerland	2019-2020	14 health care professionals, 14 stroke survivors, and 6 family members of stroke survivors	CDSS^b^
Chen et al (2021) [13]	United Kingdom	2018-2020	12 physicians (radiologists) and 6 radiographers	Various
Huang et al (2023) [14]	Singapore and India	2022	45 physicians	CDSS
Jussupow et al (2022) [15]	Germany	2017-2019	164 medical students and 42 medical professionals	CDSS
Kocaballi et al (2020) [16]	Australia	NR	16 physicians (GPs^c^)	DA^d^
Lombi and Rossero (2023) [17]	Italy	2021	12 physicians (radiologists)	Various
Wong et al (2023) [18]	China	2021	16 physicians (PCPs^e^) and 24 patients	DDS^f^

^a^AI: artificial intelligence.

^b^CDSS: clinical decision support systems.

^c^GP: general practitioner.

^d^DA: documentation assistant.

^e^PCP: primary care physician.

^f^DDS: digital disease surveillance.

### Conceptualizing and Assessing Physician Autonomy

The studies differed significantly in how they conceptualized physician autonomy and to what extent physician autonomy was the focus of their research. In particular, only 1 study [17] explicitly named (the effect of AI on) physician autonomy as a research focus (Table 2). The remaining studies focused on expectations and acceptance of or views and attitudes toward AI.

**Table 2:** The role of physician autonomy in the included studies.

	[12]^a^	[13]	[14]	[15]	[16]	[17]	[18]
Physician autonomy is an explicit focus of the study						✓	
Questions explicitly include physician autonomy			✓			✓	
Questions implicitly include physician autonomy	✓		✓		✓	✓	

^a^The interview questions reference “autonomy,” but not explicitly physician autonomy.

Only 2 of 7 included studies [14,17] explicitly included physician autonomy in their interview, survey, or focus group questions, and of these, only one study [17] uses a concrete theoretical framework for physician autonomy. Nevertheless, more than half of the studies (implicitly) included at least some aspects of physician autonomy in their interview questions, even if they did not explicitly relate them to physician autonomy. The remaining studies did not include physician autonomy in their interview questions but did identify aspects of physician autonomy in their participants’ responses. Therefore, although most studies did not explicitly name physician autonomy as a research focus or in their interview questions, the qualitative results of all studies include a number of themes related to physician autonomy. We categorized these results into the 7 components of physician autonomy proposed by Schulz and Harrison [5] and formed 2-3 subcomponents for each component, described in the following sections.

### Social and Economic Subcomponents of Physician Autonomy

For the choice of specialty and practice location, we identified two subcomponents: (1) AI replacing physicians and (2) AI replacing specialties. Three studies [12,15,16] reported that physicians feared becoming redundant or being replaced by AI. This represents an (indirect) threat to physician autonomy in choosing their specialty and practice location, as this choice will not be available to physicians who have been replaced by AI. In contrast, however, participants in 2 studies [12,16] argued that AI cannot or will not replace physicians, either because fully autonomous medical AI was seen as unrealistic (at least in the near future) or because AI was seen as unable to perform core tasks of (human) physicians, such as empathy and human warmth or communication.

A number of studies also addressed the risk of certain physician specialties, such as GPs [16] and radiologists [13,17], being replaced by or becoming mere assistants of AI—a direct threat to physician autonomy in choosing specialty and practice location. However, 2 studies [13,17] also found that radiologists were seen as less vulnerable to replacement by AI since their roles encompass a wide range of challenging activities (including complex diagnoses and patient relationships), which AI cannot replace as easily as routine reporting activities.

For control over the nature and volume of medical tasks, we identified three subcomponents: (1) the effect of AI on workflow and efficiency, (2) the ability of physicians to personalize and customize AI tools, and (3) involving physicians in AI design and creation. Participants in all 7 studies [12-18] believed that AI could increase efficiency by redefining workflows, taking over mundane and repetitive administrative tasks, and allowing faster decision-making. This would help address workforce shortages and free up more time for physicians to pursue other, more preferred tasks, such as research or treating complex cases. In this way, AI could enhance physician autonomy over the nature and volume of their tasks. However, participants in 3 of these studies [14,16,17] also expressed hesitation about the time-saving potential of AI, noting that additional time and effort may be required to input required data, fix errors, and train both physicians and AI systems.

Two studies [14,16] addressed further subcomponents relevant to physician control over the nature and volume of medical tasks. At the micro level, these studies addressed the ability of physicians to personalize and customize AI systems. In particular, AI systems may also enhance physician autonomy over the nature and volume of their work through personalized and adaptive features [16], although physicians in 1 study did not find AI customizability necessary [14]. At the macro level, both studies [14,16] addressed the importance of involving physicians in the design and creation of AI systems. While not every physician can be involved in the cocreation of AI, this would nevertheless increase the control of physicians as a group over the AI systems they will be working with. Table 3 shows the distribution of the components or subcomponents for social and economic freedoms among the included studies. Note that none of the included studies addressed control over earnings.

**Table 3:** Social and economic components or subcomponents of physician autonomy.

Component or subcomponent			Number of studies		Studies
**Choice of specialty and practice location**					
	AI^a^ replacing physicians	3		[12,15,16]	
	AI replacing specialties	3		[13,16,17]	
	Total	5		[12,13,15-17]	
**Control over earnings**					
	Total	0		—^b^	
**Control over the nature and volume of medical tasks**					
	AI and workflow or efficiency	7		[12-18]	
	AI customization or personalization	2		[14,16]	
	Involving physicians in AI design or creation	2		[14,16]	
	Total	7		[12-18]	

^a^AI: artificial intelligence.

^b^Not applicable.

### Clinical Subcomponents of Physician Autonomy

For control over diagnosis and treatment, we identified two subcomponents: (1) the (direct) effect of AI on clinical decision-making and (2) the effect of AI on physicians’ expertise and skills. Five studies [12-14,16,18] reported concerns that AI may negatively affect physicians’ clinical decision-making autonomy; participants in most of these studies [12-14] agreed that physicians should remain the final authority in clinical decision-making. Participants in other studies were less concerned about this risk, arguing that AI systems will not negatively affect physician autonomy when their adoption is voluntary [14] or when they are used as only one of many criteria informing physicians’ clinical decisions [17].

In contrast, 4 studies [12,14-16] reported that AI systems may enhance physician autonomy in clinical decision-making, particularly for less experienced physicians, by affirming their decisions and increasing decision certainty, providing inspiration and offering new possibilities of care, or helping clinicians adhere to guidelines (note that while Amann et al [12] describe better adherence to guidelines as a positive effect of AI, a close reading of Schulz and Harrison [5] suggests that strict adherence to guidelines may, in fact, decrease physician control over diagnosis and treatment).

All but 1 study [12,14-18] addressed the risk of automation bias, or the overreliance of physicians on AI systems, particularly when the use of such systems is mandated [14]. In addition to diagnostic errors [17], this overreliance may lead to deskilling and loss of expertise, especially in younger generations of physicians [12,14], indirectly reducing physicians’ control over diagnosis and treatment by making some courses of action unavailable. Participants in 2 studies [13,17], however, were less concerned about this risk. For example, radiologists in 1 study [13] argued that their wide array of high-level tasks made them less vulnerable to deskilling by AI.

Conversely, 4 studies [12,13,15,16] found that AI systems may enhance the expertise and skills of physicians, thereby increasing rather than decreasing their control over diagnosis and treatment. For example, AI may assist physicians who are struggling to be empathetic by suggesting empathetic statements [16] or providing relevant and up-to-date information, especially for novice physicians [15].

Concerning control over the evaluation of care, we identified two subcomponents: (1) the effect of AI on the risk of medicolegal consequences for physicians and (2) the effect of AI on evaluations of care by patients. All but 1 study [12-17] addressed the risk of medicolegal consequences resulting from the use of AI systems. On the one hand, physicians feared the liability issues that may arise from disagreeing with AI decisions or recommendations [15,16], particularly in light of potential data biases in AI systems. On the other hand, they feared that AI systems may be used as auditing tools [16], retrospectively assessing physician’s consultation and treatment records for potential errors in diagnosis or treatment. While many study participants agreed that the responsibility—and liability—for medical decisions involving AI rests with physicians as the final decision makers [12,14,17], a number of participants suggested that other actors, such as developers [12], host units [13], or hospitals [14], could share this responsibility (in full or in part).

Five studies [12,14-16,18] addressed the effects of AI on patient evaluations of care. On the one hand, participants in most of these studies feared that patients would negatively react to the use of AI because dependence on AI may undermine patients’ faith in the competence of physicians and their recommendations [15,16], because intransparency about AI’s use of patient data may threaten patient trust in physicians [18] or because patients may simply prefer human physicians [14]. On the other hand, some studies suggested that patients may approve of the use of AI as an evidence-based approach that can lead to improved care outcomes [14,15], and while Amann et al [12] found that patients should have a say when it comes to the use of AI, Huang et al [14] found that many physicians felt it unnecessary to discuss AI use with patients.

Finally, we identified two subcomponents for control over other professionals: (1) indirect control and (2) direct control, which were addressed by two studies each. Indirect control refers to the status and prestige of physicians (individually and as a profession) in relation to other professionals, including other physicians. While Jussupow et al [15] found that AI systems were seen as leading to a loss in status and prestige for physicians in general, Lombi and Rossero [17] suggested that the advent of AI may present an opportunity for radiologists to reconfigure their professional identity and actually increase their status and prestige by becoming proficient in these technologies.

Direct control refers to the ability of physicians to directly influence or exercise authority over other professionals, including other physicians. While 2 studies [14,17] addressed this component, they conceptualized the effect of AI on professional control in different ways and no overarching themes emerged between them. On the one hand, Huang et al [14] found that senior physicians would encourage junior physicians to use AI and that physicians would, in fact, be influenced by colleagues to adopt AI. On the other hand, Lombi and Rossero [17] found that AI may transform and expand radiologists’ interprofessional collaboration (including with nonclinical professionals). AI was seen as threatening professional boundaries and risking a loss of radiologist authority to other clinical professionals but was not seen as challenging radiologists’ professional boundaries or authority concerning nonclinical professionals [17]. Table 4 shows the distribution of the components or subcomponents for clinical freedoms among the included studies. Note that none of the included studies addressed the acceptance of patients.

**Table 4:** Clinical components or subcomponents of physician autonomy.

Component or subcomponent			Number of studies		Studies
**Acceptance of patients**					
	Total	0		—^a^	
**Control over diagnosis and treatment**					
	AI^b^ and clinical decision-making	7		[12-18]	
	AI and physician expertise or skills	7		[12-18]	
	Total	7		[12-18]	
**Control over the evaluation of care**					
	AI and medicolegal consequences	6		[12-17]	
	AI and patient evaluations of care	5		[12,14-16,18]	
	Total	7		[12-18]	
**Control over other professionals**					
	AI and indirect control over other professionals	2		[15,17]	
	AI and direct control over other professionals	2		[14,17]	
	Total	3		[14,15,17]	

^a^Not applicable.

^b^AI: artificial intelligence.

### Potential Benefits and Harms of AI for Physician Autonomy

The main results of the included studies in each subcomponent are summarized in Textboxes 4 (for social and economic freedoms) and 5 (for clinical freedoms). For 6 of 11 subcomponents, we found mixed results concerning the potential benefits and harms of AI for physician autonomy. In particular, studies disagreed on whether AI will increase or decrease workflow efficiency, enhance or impede clinical decision-making, improve or worsen physician skills and expertise, lead to patient approval or disapproval, and increase or decrease physician status or prestige. Studies were also split on how AI will affect physicians’ direct control over other professionals.

**Potential benefits and harms of artificial intelligence (AI) for social and economic freedoms, indicated by (+) and (–), respectively. Circles indicate relevant findings that are neither harms nor benefits.**

**Choice of specialty and practice location**

AI replacing physicians (n=3)

- (+) AI (currently) lacks the capabilities, such as empathy, necessary to replace physicians
- (−) AI may replace physicians in the future

AI replacing specialties (n=3)

- (+) Radiologists are less vulnerable to AI replacement due to their wide range of challenging activities
- (−) AI may replace radiologists in the future
- (−) AI may replace general practitioners in the future

**Control over the nature and volume of medical tasks**

AI and workflow or efficiency (n=7)

- (+) AI can increase efficiency by handling mundane activities, freeing up time for other tasks
- (−) AI may decrease efficiency due to the time and effort required for data input, error correction and training

AI customization or personalization (n=2)

- (+) AI may support physicians through personalized and adaptive features

Involving physicians in AI design or creation (n=2)

- (o) Physicians should be involved in AI design or creation

For 2 subcomponents (AI replacing physicians and AI replacing specialties), we found mixed to negative results. On the one hand, the studies that addressed these 2 components found that physicians and some specialties (radiologists and GPs or primary care physicians) may be at risk of replacement by AI. On the other hand, the studies gave a number of reasons why physicians and some specialties may be less vulnerable to such replacement, at least in the near future. However, while these results are not fully negative, we did not find any results indicating that AI may improve physician autonomy in these subcomponents.

**Potential benefits and harms of artificial intelligence (AI) for clinical freedoms, indicated by (+) and (–), respectively. Circles indicate relevant findings that are neither harms nor benefits.**

**Control over diagnosis and treatment**

AI and clinical decision-making (n=7)

- (+) AI may enhance clinical autonomy by increasing decision certainty and providing inspiration
- (−) AI may harm clinical decision-making autonomy
- (o) Physicians should remain the final authority in clinical decision-making

AI and physician expertise or skills (n=7)

- (+) AI may enhance physicians’ expertise
- (−) AI may lead to loss of expertise through overreliance and automation bias

**Control over evaluation of care**

AI and medicolegal consequences (n=6)

- (−) AI decisions and recommendations may lead to liability issues for physicians
- (−) AI systems may be used as post hoc auditing tools
- (o) Developers, hospitals, or other actors should (partially) share responsibility for medical decisions involving AI

AI and patient evaluations of care (n=5)

- (+) Patients may approve of AI use (eg, due to improved outcomes)
- (−) AI may lead to patient disapproval or mistrust
- (−) AI may undermine patients’ faith in physicians’ care

**Control over other professionals**

AI and indirect control over other professionals (n=2)

- (+) AI may offer radiologists an opportunity to increase their status and prestige
- (−) AI systems may lead to a loss in status and prestige for physicians in general

AI and direct control over other professionals (n=2)

- (+) AI may expand radiologists’ interprofessional collaboration with nonclinical professionals
- (−) AI may threaten radiologists’ authority over other clinical professionals
- (−) Physicians may be influenced by peers and superiors to adopt AI

In contrast, we found general agreement between the included studies for the remaining 3 subcomponents. For AI customization or personalization, this consensus was positive: both studies addressing this component found that customizable AI systems would support physician autonomy. Furthermore, there was agreement between studies that AI represented potential harms (but not benefits) to physician autonomy in the AI and medicolegal consequences component. Finally, both studies that addressed involving physicians in AI design or creation found that such involvement should take place (although this more accurately represents a recommendation or demand rather than a potential benefit or harm).

## Discussion

### Principal Results

These results show that research on the potential effects of AI on physician autonomy is still in its nascency. In particular, there is no consensus definition or operationalization of physician autonomy in qualitative research. Most studies did not name physician autonomy as a focus of their research or explicitly include physician autonomy in their interview, survey, or focus group questions. In fact, only 1 study [17] specified a clear theoretical framework for physician autonomy. These results align with existing research on the professional autonomy of nurses, which has been found to face challenges due to inconsistent definitions and inappropriate measures of nurse autonomy [19] and the confounding of the clinical and nonclinical aspects of nurse autonomy [20].

No studies addressed a complete set of components of physician autonomy (as defined by Schulz and Harrison [5]). Furthermore, coverage between components varies significantly: while all 7 studies addressed control over the nature and volume of medical tasks, control over diagnosis and treatment, and control over the evaluation of care, none of the included studies addressed control over earnings and acceptance of patients.

We identified a total of 11 subcomponents for the 5 components of physician autonomy that were addressed by at least 1 study. For most of these subcomponents, studies reported mixed results concerning the potential harms and benefits of AI for physician autonomy. A notable exception addressed by most studies was AI and medicolegal consequences*,* with studies reporting only potential harms for this subcomponent. AI customization or personalization was the only subcomponent in which only potential benefits were reported, although this subcomponent was only addressed by 2 studies. Overall, there is a need for further research that focuses specifically on physician autonomy and includes a full conception of its components and subcomponents.

Some of the results within subcomponents align with recent reviews of the academic literature, which have found positive effects of AI on clinical and administrative workflow or efficiency or patient-physician trust [21,22]. A recent review of the “grey literature” also found that clinical and administrative AI applications impact physician job autonomy, skills, and professional relationships [23]. However, not all of these results are reported by the reviews as components of physician autonomy.

### Limitations

However, the methodological limitations of our scoping review should be considered when interpreting our results. In particular, we identified only 7 studies that fit the inclusion criteria. Furthermore, although 4 of 7 studies [12,14,17,18] were published in 2023, only 1 study [14] specified a data collection period later than 2021 and 3 studies completed their data collection before the end of 2020. Considering the rapid evolution of AI in medicine, such as the recent introduction of large language models such as ChatGPT [24,25], there is a clear need for additional, up-to-date research on physician autonomy and new AI systems.

Furthermore, we included only qualitative studies in this review. In our view, expanding our scope to include a full systematic review of quantitative studies on AI and physician autonomy would have been premature, as the field is comparatively new and because we were focused particularly on how physician autonomy is defined and conceptualized by researchers and participants. However, the subcategories we have identified provide a useful roadmap for future systematic reviews of quantitative studies on physician autonomy and AI, and such reviews should be conducted.

Our review may also have missed further studies that were not included in the databanks we searched or that did not explicitly mention (physician) autonomy. However, these studies may still be relevant: while we assigned study results to components of physician autonomy in order to form inductive subcomponents, most of the included studies do not conceptualize physician autonomy as covering each of these components. For example, subcomponents such as AI and workflow or efficiency*,* AI and physician expertise or skills, or AI and patient evaluations of care were addressed by a number of studies, but usually not explicitly related to physician autonomy. This indicates that there may be further studies that address relevant components without explicitly mentioning autonomy. This should also be considered when conducting future systematic reviews of quantitative studies on physician autonomy and AI. In particular, search terms related to specific subcomponents (but not physician autonomy) may lead to the inclusion of additional relevant studies.

Future research should also explicitly include the 2 components that were not addressed by any of the studies in our review: control over earnings and acceptance of patients. In particular, one should not conclude from our review that AI will have no effect on physician autonomy for these components. Such a conclusion seems implausible since examples of possible effects are easily constructed. For example, if AI systems were to take on the role of gatekeepers and play some part in deciding which patients can be seen by which physicians, this would represent harm to physician autonomy. Instead, the absence of these components from our review should be taken to indicate that respondents (or researchers) did not conceive of control over earnings and acceptance of patients as (relevant) aspects of physician autonomy.

Studies also differed in their definition of AI, which complicates evidence comparison and synthesis. While some studies considered AI-based CDSS, others considered different AI systems or AI innovations more broadly, and while 1 study [17] recruited participants who had actual working experience with AI systems, most merely presented participants with vignettes describing possible AI systems. This means that most studies report only the potential harms and benefits of AI (as feared or hoped for by participants), not actual harms and benefits. As a systematic comparison of the effects of different types of AI systems on physician autonomy was not possible with only 7 included studies, our scoping review is further limited to a broader discussion of the potential effects of AI in general. However, further research should analyze these differences in effect, based (where possible) on evaluations of actual AI systems, rather than vignettes.

Initial evidence also suggests that participants in different regions or cultures perceive different potential harms and benefits of AI for physician autonomy. For example, Huang et al [14] found that views on (the effects of AI on) some aspects of physician autonomy differed between physicians in Singapore and India, while Wong et al [18] discuss the fragility of doctor-patient trust specifically in China. While we were unable to analyze these differences due to the limited number of studies, future research should more thoroughly investigate such cultural and geographic differences in attitudes toward both AI and physician autonomy.

Overall, our results are based on a limited number of studies and should be seen as opening, rather than closing, lines of inquiry into the effects of AI on physician autonomy. Fully understanding these effects will require an ambitious research program. First, there is a need for further qualitative studies focusing explicitly on physician autonomy. Second, a definitive understanding of AI and physician autonomy will require quantitative studies using validated and reliable instruments designed for this purpose. Finally, the current literature focuses almost exclusively on self-reported physician autonomy. However, it may also be possible to measure the effect of AI on physician autonomy using objective quantitative indicators, such as the number of alerts and reviews triggered by AI systems or test results from experimental studies of physician expertise. Future research should consider if and when the use of such indicators in addition to self-reported assessments of physician autonomy is appropriate.

### Conclusions

Little research to date has addressed the potential effects of AI on physician autonomy. Existing results on AI and physician autonomy are mostly secondary findings or merely part of larger analyses into physicians’ attitudes toward and acceptance of AI. Most studies addressed physician autonomy only indirectly in their research focus and interview, survey, or focus group questions.

While 3 of the components of physician autonomy proposed by Schulz and Harrison [5] were addressed by all included studies, 2 components were not addressed by any studies. In eleven (inductively formed) subcomponents, the included studies reported a number of potential effects of AI on physician autonomy. However, results were mixed, with studies reporting both potential harms and benefits of AI for physician autonomy in most subcomponents.

In conclusion, further qualitative and quantitative research is needed that focuses explicitly on physician autonomy and addresses all relevant components of physician autonomy. Where possible, research on the effects of AI on physician autonomy should be based on real experience with AI systems, rather than vignettes, and consider the differences between different AI systems and between physicians in different cultural and geographic settings.

## Appendix

Multimedia Appendix 1 PRISMA-ScR (Preferred Reporting Items for Systematic reviews and Meta-Analyses extension for Scoping Reviews) checklist.

Multimedia Appendix 2 Search terms for PubMed/MEDLINE and Web of Science.

## Abbreviations

AI: artificial intelligence
CDSS: clinical decision support system
GP: general practitioner
PRISMA: Preferred Reporting Items for Systematic Reviews and Meta-Analyses

## Data Availability

The data sets generated during or analyzed during this study are available from the corresponding author upon reasonable request.
