# Supplementary material for: Studying the Potential Effects of Artificial Intelligence on Physician Autonomy: Scoping Review
Source: JMIR AI. 2025 Mar 13;4:e59295. doi: 10.2196/59295 (PMC11950692; doi:10.2196/59295)
Supplement: Multimedia Appendix 2 [file ai_v4i1e59295_app2.doc]

**Search terms** PubMed:

|  | **P** | **I** | **C** | **O** | **S** |
| --- | --- | --- | --- | --- | --- |
| **MeSH** | “Physicians”  “Physicians, Primary Care”  “General Practitioners”  “Hospitalists” | “Artificial Intelligence”  “Deep Learning”  “Machine Learning” |  | “Professional Autonomy” | “Qualitative Research” |
| **Title/Abstract** | “Physician”  “Physicians”  “Doctor”  “Doctors”  “Clinician”  “Clinicians”  “Hospitalist”  “Hospitalists”  “General Practitioner”  “General Practitioners”  “Medical Profession”  “Medical Professions” | “AI”  “Artificial Intelligence”  “ML”  “Machine Learning”  “Machine-based Learning”  “Deep Learning”  “DL” |  | “autonomy”  “autonomies”  “autonomous”  “autonomical”  “autonomic”  “autonomously”  “autonomically” | “Qualitative”  “Focus groups”  “Interview”  “Interviews” |

**Search terms Web of Science**:

|  | **P** | **I** | **C** | **O** | **S** |
| --- | --- | --- | --- | --- | --- |
| **Topics** | “Physician”  “Physicians”  “Doctor”  “Doctors”  “Clinician”  “Clinicians”  “Hospitalist”  “Hospitalists”  “General Practitioner”  “General Practitioners”  “Medical Profession”  “Medical Professions” | “AI”  “Artificial Intelligence”  “ML”  “Machine Learning”  “Machine-based Learning”  “Deep Learning”  “DL” |  | “autonomy”  “autonomies”  “autonomous”  “autonomical”  “autonomic”  “autonomously”  “autonomically” | “Qualitative”  “Focus groups”  “Interview”  “Interviews” |
